# Novel gene loci associated with susceptibility or cryptic quantitative resistance to *Pyrenopeziza brassicae* in *Brassica napus*

**DOI:** 10.1007/s00122-023-04243-y

**Published:** 2023-03-23

**Authors:** Heather Fell, Ajisa Muthayil Ali, Rachel Wells, Georgia K. Mitrousia, Hugh Woolfenden, Henk-jan Schoonbeek, Bruce D. L. Fitt, Christopher J. Ridout, Henrik U. Stotz

**Affiliations:** 1 Centre for Agriculture, Food and Environmental Management, School of Life and Medical Sciences, University of Hertfordshire, Hatfield, AL10 9AB UK; 2 Crop Genetics Department, John Innes Centre, Norwich Research Park, Norwich, NR4 7UH UK; 3 Computational and Systems Biology, John Innes Centre, Norwich Research Park, Norwich, NR4 7UH UK; 4 Present Address: Communication and Engagement Office, Science Innovation Engagement Partnerships, Rothamsted Research Ltd, West Common, Harpenden, AL5 2JQ UK

## Abstract

**Key message:**

Quantitative disease resistance (QDR) controls the association of the light leaf spot pathogen with *Brassica napus*; four QDR loci that were in linkage disequilibrium and eight gene expression markers were identified.

**Abstract:**

Quantitative disease resistance (QDR) can provide durable control of pathogens in crops in contrast to resistance (*R*) gene-mediated resistance which can break down due to pathogen evolution. QDR is therefore a desirable trait in crop improvement, but little is known about the causative genes, and so it is difficult to incorporate into breeding programmes. Light leaf spot, caused by *Pyrenopeziza brassicae*, is an important disease of oilseed rape (canola, *Brassica napus*). To identify new QDR gene loci, we used a high-throughput screening pathosystem with *P. brassicae* on 195 lines of *B. napus* combined with an association transcriptomics platform. We show that all resistance against *P. brassicae* was associated with QDR and not *R* gene-mediated. We used genome-wide association analysis with an improved *B. napus* population structure to reveal four gene loci significantly (*P* = 0.0001) associated with QDR in regions showing linkage disequilibrium. On chromosome A09, enhanced resistance was associated with heterozygosity for a cytochrome P450 gene co-localising with a previously described locus for seed glucosinolate content. In addition, eight significant gene expression markers with a false discovery rate of 0.001 were associated with QDR against *P. brassicae*. For seven of these, expression was positively correlated with resistance, whereas for one, a HXXXD-type acyl-transferase, negative correlation indicated a potential susceptibility gene. The study identifies novel QDR loci for susceptibility and resistance, including novel cryptic QDR genes associated with heterozygosity, that will inform future crop improvement.

**Supplementary Information:**

The online version contains supplementary material available at 10.1007/s00122-023-04243-y.

## Introduction

Quantitative disease resistance (QDR) is predominant in natural plant populations and provides robust and durable protection from pathogens in ecosystems (Delplace et al. 2020). Although QDR could potentially provide more durable resistance in crop plants, it is not routinely selected for in breeding since the underlying gene loci are poorly characterised and contribute partial but additive resistance which is difficult to track in breeding programmes (Nelson et al. 2018). Understanding the molecular basis of QDR will provide novel opportunities for introducing durable disease resistance into crops and reducing the use of pesticides. Our investigation uses a pathosystem which, combined with an association genetics platform, establishes a novel approach to identify candidate QDR genes for crop improvement in *Brassica napus*.

Plants have evolved layers of immunity for defence against various pathogens with different modes of infection and life styles. Although the layers of plant immunity are not distinct and may overlap to some extent (Ngou et al. 2021; Yuan et al. 2021), the first is considered to be the rapid detection of microbial patterns usually described as pattern-triggered immunity (PTI). Adapted pathogens secrete effectors that suppress PTI as they colonise the host plant and cause disease (Jones and Dangl 2006). Effectors can be recognised by resistance proteins, typically nucleotide-binding, leucine-rich repeat (NLR) immune receptors, which provide the second layer of immunity to protect the plant. When operating against cell-penetrating pathogens, this effector-triggered immunity (ETI) is the principal mechanism in qualitative, or *R* gene-mediated, resistance and provides a powerful defence response (Jones & Dangl 2006). However, operating against extracellular pathogens, *R* gene-mediated resistance, termed effector-triggered defence (ETD) acts more slowly and is not an immune but a resistance response (Stotz et al. 2014). Pathogen populations can mutate or lose effectors so that they are no longer recognised, leading to resistance breakdown in crops. This contrasts with QDR, which is predicted to provide durable protection against pathogens. Although less well-studied, mechanisms of QDR can include enhanced cell wall thickening, modified signalling processes and enhanced secondary metabolism (Cowger and Brown 2019). Interestingly, studies with the apoplastic (extracellular) fungal pathogen *Leptosphaeria maculans* have shown that ETD may also contribute to QDR in *B. napus*, reinforcing the concept of overlap between layers of defence in plants (Jiquel et al. 2021).

After soybean, oilseed rape (OSR; *Brassica napus*) is economically the second most important vegetable oil crop in the world. Amongst the biotic threats that challenge OSR production, light leaf spot (LLS), caused by the apoplastic ascomycete *Pyrenopeziza brassicae* (anamorph *Cylindrosporium concentricum*), ranks in the top 10 most damaging diseases on the crop in Europe (Zheng et al. 2020); note that *P. brassicae* is the perfect stage of *C. concentricum*. Yield losses are due to seedling death at the rosette stage, stunting of susceptible cultivars and floral infection, leading to malformed pods and seeds, premature pod senescence and pod shattering prior to harvest (Gilles et al. 2000). This pathogen has a widespread geographic distribution, occurring in the United Kingdom (UK) and, increasingly, continental Europe, the Pacific Northwest of the United States, Asia (Japan, Philippines) and New Zealand (Carmody et al. 2019; Karandeni Dewage et al. 2018). *P. brassicae* epidemics are initiated by wind-dispersed ascospores. Acervuli then produce asexual conidia in infected plant parts; these conidia are rain splash-dispersed to establish the polycyclic stage of the LLS disease epidemic.

LLS has become the most damaging disease of OSR in the UK. The disease accounts for up to £160 million yield loss annually in England, despite expenditure of £20 M on fungicides. The severity of the disease is greater in Scotland than in England (Karandeni Dewage et al. 2018). However, LLS has progressively become a greater problem in parts of the UK other than Scotland over more than a decade and may now be considered a national emergency. Simultaneously, *P. brassicae* has become a problem on Brassica vegetables in the UK and other places, causing market losses due to surface blemishes on crops like Brussels sprouts. Besides *B. napus* and *B. oleracea*, *P. brassicae* has been observed on mustard rape (*B. juncea*) and *B. rapa* (Carmody et al. 2019).

One of the limitations of studying QDR in crop species is that resistance of individual QDR loci is only partial and can be masked by *R*-gene-mediated resistance. *P. brassicae* on *B. napus* is an ideal pathosystem to study QDR because there are no known *R* genes effective against the pathogen. Moreover, one study has indicated the presence of QDR for LLS. Six environmentally stable quantitative trait loci (QTL) for resistance against *P. brassicae* were mapped in a doubled haploid (DH) population of *B. napus* derived from a cross between moderately resistant Darmor-*bzh* and susceptible Yudal cultivars (Pilet et al. 1998). Four new QTL for resistance against *P. brassicae* were identified on linkage groups C01, C06 and C09 (Karandeni Dewage et al. 2022). Two major loci for resistance against *P. brassicae* were identified (Bradburne et al. 1999), and one was mapped to the bottom of chromosome A1 of *B. napus* (Boys et al. 2012). This locus could be a QDR locus since it was associated with substantial decrease in LLS hyphal growth rather than death of the pathogen typical of *R* gene-mediated resistance (Boys et al. 2012; Stotz et al. 2014). However, apart from these examples, little is known about QDR for resistance against *P. brassicae* in *B. napus*.

*P. brassicae* and *Rhynchosporium commune* are closely related discomycetes (Goodwin 2002; Penselin et al. 2016) that occupy a subcuticular apoplastic niche in their respective brassica and barley hosts (Stotz et al. 2014). It is therefore relevant to compare resistance mechanisms that operate against these related pathogens. The major resistance locus *Rrs1*, containing wall-associated kinases, controls resistance against *Rhynchosporium commune* in barley (Looseley et al. 2020). *Rrs1* is the only *R* gene against *R. commune* that has a corresponding *Avr* gene, *NIP1*, that encodes a necrosis-inducing protein (Rohe et al. 1995). Additionally, multiple quantitative resistance loci were found to be involved in resistance of barley against *R. commune* (Buttner et al. 2020).

The development of association genetics and transcriptomics has enabled the identification of pathogen resistance loci that was not possible in biparental mapping populations (Bartoli and Roux 2017). The approaches take advantage of recombination events that have accumulated in natural populations to identify genetic polymorphisms associated with phenotypes of interest. The method requires genomic or transcriptomic sequences from a diversity collection and phenotypic data for the trait of interest. The approach has been used to identify new resistance loci operating against *Sclerotinia sclerotiorum* stem rot and potential *R* genes for resistance in *B. napus* against *Plasmodiophora brassicae* (clubroot) (Hejna et al. 2019; Wu et al. 2016). However, no such studies have been done to identify QDR against *Pyrenopeziza brassicae*.

The aim of this study was to characterise genomic regions associated with QDR against *P. brassicae* in *B. napus* under glasshouse conditions. To achieve this, we performed association genetic analysis with a *B. napus* diversity set developed through the OREGIN initiative (https://www.herts.ac.uk/oregin) combined with genotype and expression data (Havlickova et al. 2018) and phenotypic measurement of *P. brassicae* infection that we developed for the study. This study provides new insights into QDR mechanisms and supports breeding efforts to generate durable disease-resistant crops.

## Methods

### Glasshouse growth conditions

Glasshouses at the Bayfordbury campus, University of Hertfordshire, and Rothamsted Research were utilised for experiments scored for partial resistance against *P. brassicae* with baseline temperatures set to 16 °C during the day and 14 °C during the night (see diurnal cycles below). Actual temperatures recorded were outside this baseline range, however, due to fluctuations in temperature and light during the experimental period (Supporting Information Table S1). At Bayfordbury, supplemental lighting was used for 12 h per day using sodium high pressure lamps (Sylvania SHP-TS 400 W GroLux), which automatically switched on once natural daylight decreased to < 115 µmol m^−2^ s^−1^. Supplemental lighting (LEDs, 175 W m^−2^) was used for 12 h and 14.5 h at Rothamsted Research for experiments 8 and experiments 9 and 10, respectively (Supporting Information Table S1). The intensity of supplemental lighting was 200 µmol m^−2^ s^−1^. Humidity levels were variable as they cannot be controlled in a glasshouse situation, although they were monitored at Rothamsted Research.

### Isolation of *P. brassicae* populations

Two populations of *P. brassicae* were used. The first population was amplified having obtained infected leaves from KWS SAAT SE & Co. KGaA (Einbeck, Germany) from field experiments on the island in Fehmarn, Germany (54.4701, 11.1329) in 2016. The second population was obtained from infected leaves of KWS-grown *B. napus* genotypes, including reference cultivars Cuillin and Express, at a Rothamsted Research field site (51.813125, − 0.382005) in 2019. Populations obtained in 2016 and 2019 were used for seven and three glasshouse experiments at Bayfordbury and Rothamsted Research, respectively (Supporting Information Table S2).

Leaves of *B. napus* with the greatest amount of *P. brassicae* sporulation already present were sampled from the field experiments and placed into polyethylene bags for transportation back to the University of Hertfordshire. The spores were dislodged from the leaves by pipetting sterile distilled water over the leaf, and the run-off was collected in a glass beaker. This spore suspension was filtered using Miracloth (Merck Millipore, Watford, U.K.) and the spore concentration determined using a haemocytometer slide under a stereo-microscope (GX Microscopes, XTC-3A1). The concentration was adjusted to 10^5^ spores ml^−1^ and dispensed into 50 ml aliquots for use as inoculum in the experiments or stored at − 20 °C.

### Quantification of *P. brassicae* sporulation on leaves of *B. napus* accessions

Each of 10 experiments tested 24 accessions, each with five inoculated replicates with the exception of experiments 1 and 3, which each tested only 23 accessions (Supporting Information Table S2). In total, there were 195 accessions in these experiments. The replicates were arranged in a randomised alpha block design, so that each block included one replicate plant from each of the 24 accessions. All of the lines screened could not be grown at the same time; therefore, seven experiments were done at Bayfordbury from 2016 to 2018 with the cultivars Tapidor, Imola (resistant), Bristol (susceptible) and Temple included in each experiment to act as references for normalising symptom severity between experiments. In 2019, another three experiments were done at Rothamsted Research with the susceptible one of the replicated reference cultivars being Cabriolet instead of Bristol due to seed availability.

Seeds were stratified for 2 days at 4 °C prior to germination on damp filter paper in Petri dishes in the dark. The successfully germinated seeds were then planted into 8 × 5 cell seed trays with a 50:50 mixture of John Innes No.3 compost and multipurpose compost (Miracle Gro). The seedlings were left to grow in the glasshouse for approximately 2 weeks until the first and second true leaves had emerged. Established plants were transplanted into 9 cm diameter individual pots using the same potting mix. Additional fertiliser was not used. Seedlings were irrigated from below daily using capillary matting. Once potted, plants were checked each day and top-watered when the first 2.5 cm of soil was dry.

After a further 2 weeks, a total of 120 plants at growth stage 1,5 (Sylvester-Bradley 1985), i.e. seedlings with five true leaves, were sprayed with 100 ml of *P. brassicae* spore suspension, at a concentration of 10^5^ spores ml^−1^ water containing 0.05% Silwet (Spiess-Urania Chemicals GmbH, Hamburg, Germany) as a wetting agent. A hand sprayer on fine mist setting was used to sufficiently cover all leaves of all plants. Subsequently, plants were incubated for 48 h under a clear polyethylene sheet over a plastic frame, 2 × 1 × 0.5 m in dimension. The plants were grown for another 3 weeks before the whole plant was sampled by cutting at the base of the stem just above the soil surface, wrapped in a paper towel, and placed into a labelled polyethylene freezer bag to preserve the whole plant. As all plants were harvested at the same time, growth stages slightly varied amongst different lines. On average, plants reached growth stage 1,9. The inside of the bag and the paper towel were sprayed with sterile distilled water to create humidity, and then, sampled plants were incubated at 4 °C in a cold room. After 5 and 10 days of incubation, the sampled plants were assessed visually for the presence of *P. brassicae* sporulation using a modified LLS 1–6 severity scale (Karandeni Dewage et al. 2021). Specifically, score = 1 (no sporulation), score = 2 (< 10% leaf area with sporulation), score = 3 (10–25% leaf area with sporulation), score = 4 (25–50% leaf area with sporulation), score = 5 (50–75% leaf area with sporulation) and score = 6 (75–100% leaf area with sporulation). Each of three to six leaves per plant was given a score, and then, these scores were averaged to give a disease severity rating.

### Statistical analysis of phenotypic traits

The quantitative resistance work consisted of 10 glasshouse experiments. A total of 195 accessions were scored for *P. brassicae* sporulation. Reference genotypes replicated in each of the ten experiments were used to determine normality of the data with the Shapiro–Wilk test and lack of effect of experiment for the entire study. Phenotypic data were analysed in R using a package for nonlinear mixed-effects models (nlme). The function for the model had the following structure: model = lme(Score ~ Genotype, data = LLS, random =  ~ 1|Experiment/Genotype). Adjusted means were generated and ordered according to the scores of the genotypes. A Wilcoxon signed-rank test was used for pair-wise comparisons of the adjusted means of genotypes. A significance threshold of *P* < 0.05 was used to assess pair-wise differences in disease score. Data were visualised in R using ordered means dotchart and hist functions.

### Association transcriptomics

The phenotyping datasets of the diversity set for LLS resistance scores were analysed using an association transcriptomics pipeline based on programs used to map traits in *B. napus* previously with minor modifications (Harper et al. 2012; Wells et al. 2013). Genotype and expression level datasets used were published (Havlickova et al. 2018) and available from York Knowledgebase (http://yorknowledgebase.info). This dataset was reduced to include only the accessions within this study.

Gene expression marker (GEM) associations were determined by linear regression using Reads Per Kilobase of transcript, per Million mapped reads (RPKM) to predict a quantitative outcome of the trait value. All markers with an average expression less than 0.5 RPKM were removed before analysis.

An updated population structure was calculated for the accessions used within this study using a Bayesian clustering approach. A Markov Chain Monte Carlo (MCMC) algorithm was implemented in the population-genetic software STRUCTURE V2.3.1. One of the requirements of STRUCTURE is unlinked markers; therefore, the single nucleotide polymorphism (SNP) file was adjusted before the analysis using the following criteria: SNPs were required to be biallelic, with a minor allele frequency (MAF) > 0.05 and a minimum distance of 500 kb between markers. Markers within 100 kb of the centromeres, based on published findings (Cheng et al. 2013; Mason et al. 2016), were excluded. STRUCTURE was run using the admixture model with uncorrelated allele frequencies with a burn-in period of 100,000 iterations and MCMC analyses of 100,000 permutations. The accessions were not assigned to a given population. Ten iterations were run for each value of K, the number of subpopulations estimated to make up the total population. STRUCTURE HARVESTER (Earl and vonHoldt 2012) was used to determine the optimal *K* value, by generating a series of Δ*K* values, which represent the mean likelihood of *K* divided by the standard deviation of *K*, for the population. To further investigate population clusters, TASSEL v5 was used to construct a phylogenetic tree, using the Neighbour Joining method and all SNPs with MAF > 0.05. A cluster matching and permutation program (CLUMPP) was used from STRUCTURE HARVESTER for *K* = 6 to generate the Q matrix input (Jakobsson and Rosenberg 2007).

Genome-wide association (GWA) mapping was performed using TASSEL v5 using SNP markers with an allele frequency > 0.05. Analysis was conducted using generalised linear models (GLM) and mixed linear models (MLM) to determine the optimal model. Kinship data were calculated using TASSEL’s ‘centered IBS’ method. Optimum compression level and P3D variance component estimation were used as MLM options. The false discovery rate (FDR) was determined using the Shiny implementation of the q-value R package (Storey et al. 2020). GEM and GWA results were visualised using R (https://github.com/BRAVO-research-project/pyrenopeziza-resistance). The most significantly associated SNP markers were selected for further analysis, including distribution within the population and allelic effect.

The level of linkage disequilibrium (LD) varies between and across chromosomes depending on the position and level of selection. To determine the specific level of LD at each locus, the mean pair-wise *r*^*2*^ for all markers on a chromosome to each of the 11 significantly associated markers was calculated within TASSEL v5 using the site by all analysis option. Markers were considered in LD when *r*^*2*^ > 0.15.

## Results

### LLS scoring system

**Fig. 1:**
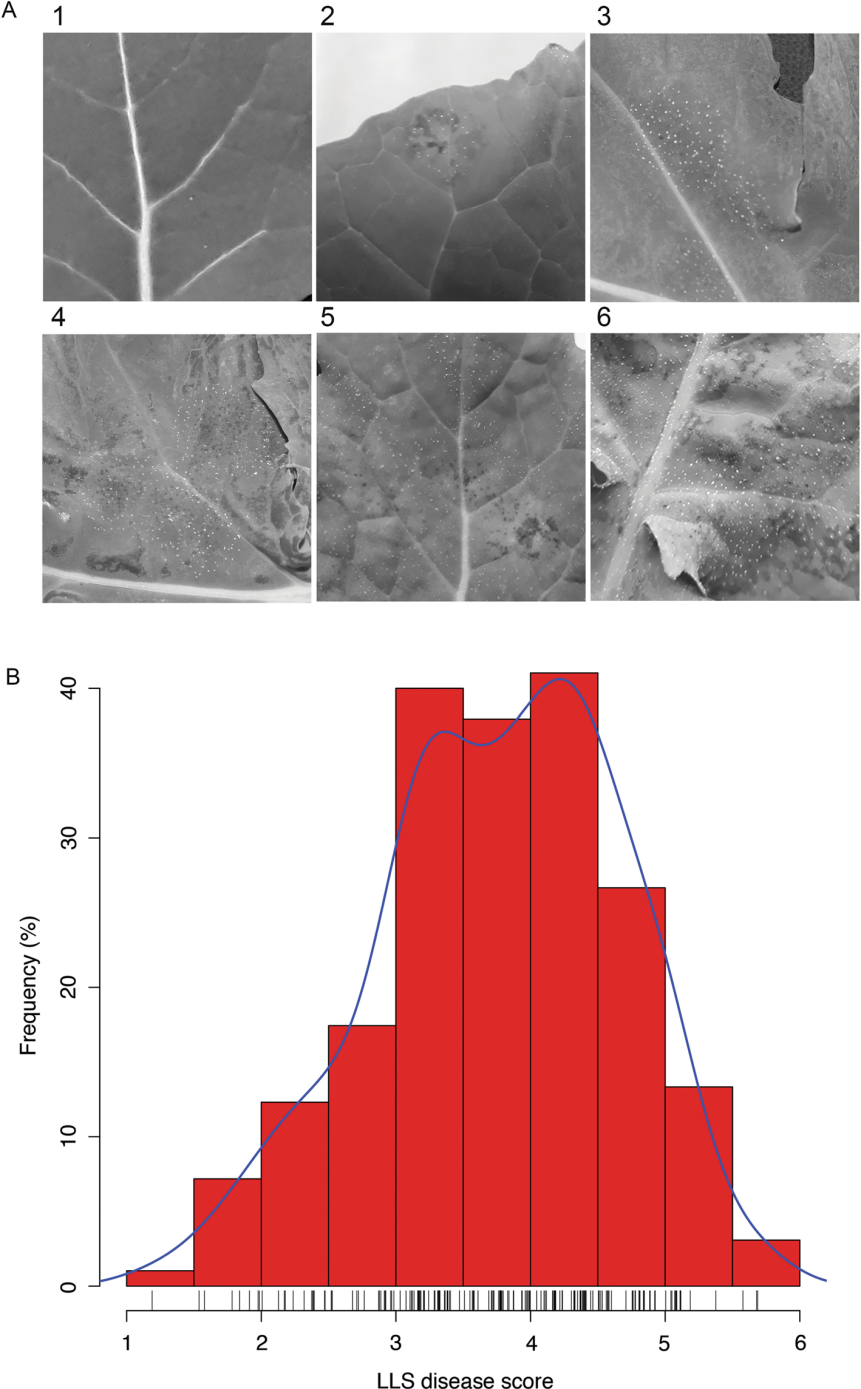
Phenotypic assessment of *P. brassicae* sporulation on *B. napus* leaves, following 10 days of incubation at high humidity after sampling. **A** Sporulation structures (acervuli) can be visualised macroscopically. Acervuli were best visualised using greyscale images to improve contrast. The percentage leaf area with sporulation is assessed as the disease score and expressed on a scale of 1 to 6; numbers above images refer to these disease scores. Accessions/cultivars Bristol (1, no sporulation), Tapidor (2, < 10% leaf area with sporulation), ‘couve-nabiça’ (3, 10–25% leaf area with sporulation), Capitol (4, 25–50% leaf area with sporulation), Musette (5, 50–75% leaf area with sporulation) and Daichousen (6, 75–100% leaf area with sporulation) are shown. **B** Distribution of disease scores, representing the percentage leaf area with *P. brassicae* sporulation among 195 diverse *B. napus* accessions on a scale of 1 to 6. The data represent a total of 1190 assessments in 10 glasshouse experiments, each with 24 accessions and five replicates, with the exception of two experiments that contained only 23 accessions (Table S1). Note that the data are approximately normally distributed. The scores are adjusted means after analysis of a linear mixed model. Ten bins (red bars) were created for the histogram. The blue density curve is a kernel density estimate and provides a smoother description of the distribution. The rug plot below is a one-dimensional representation of adjusted means for individual accessions

Percentage of leaf area with *P. brassicae* sporulation observed on infected leaves was the most consistent measurement of disease severity across all accessions (Karandeni Dewage et al. 2021) and was therefore used as the basis for the scoring system. Disease severity was scored on a scale of 1 to 6, with a score of 1 for no sporulation and 6 for the most sporulation (Fig. 1A, Supporting Information Figure S1). The image of part of an infected cv. Tapidor leaf (Fig. 1A, score 2) shows why *P. brassicae* is referred as to *C. concentricum* in its imperfect stage; this pathogen produces concentric rings of acervuli on its hosts. Patchy sporulation was observed on leaf laminas of ‘couve-nabiça’ and cv. Capitol with scores of 3 to 4, respectively (Fig. 1A). The entire leaf laminas of cv. Musette and cv. Daichousen were covered with acervuli and scored 5 and 6, respectively.

### Variation in sporulation of *P. brassicae* on leaves of diverse *B. napus* accessions

A diversity set of 195 accessions was tested in glasshouse experiments for the amount of pathogen sporulation after spray inoculation of *B. napus* seedlings with local *P. brassicae* populations. The distribution of disease scores showed wide variation in *P. brassicae* sporulation among diverse *B. napus* accessions with an approximately normal distribution of this trait (Fig. 1B).

The glasshouse screen consisted of 10 independent experiments, each with 23 or 24 *B. napus* accessions with four reference cultivars per experiment (Supporting Information Table S2). The first seven experiments were done at the Bayfordbury campus of the University of Hertfordshire and the last three experiments at Rothamsted Research. Instead of cv. Bristol, cv. Cabriolet was used as a susceptible reference cultivar for the experiments at Rothamsted Research. All other reference cultivars were identical between both sites. Irrespective of the location, there were significant differences between reference cultivars in disease score. No significant effects of experiment and experiment-by-cultivar interaction on disease score were observed (Supporting Information Notes S1). Combination of all 10 experiments and comparison of the shared reference cultivars Imola, Tapidor and Temple resulted in significant effects of both cultivar and experiment, but no significant experiment-by-cultivar interactions on disease score (Supporting Information Table S2). The disease scores of all three reference cultivars were less at Rothamsted Research than at Bayfordbury, with cv. Temple differing the most between these two environments (Supporting Information Figure S2). Restricting the analysis to cv. Imola and cv. Tapidor eliminated the significant effect of experiment. Differences in disease scores between Rothamsted Research and Bayfordbury could have resulted from different environmental conditions, different pathogen inoculum and/or different assessors at the two sites.

Nonlinear mixed model analysis established that *B. napus* cv. Cabriolet and cv. Imola scored as the most susceptible and most resistant cultivars amongst the five reference cultivars tested, respectively (Supporting Information Table S2). Intermediate scores were observed for the other three cultivars Temple, Tapidor and Bristol. While cv. Cabriolet scored as the most susceptible cultivar amongst all 195 accessions tested, 60 accessions scored less sporulation than cv. Imola; half of these 60 accessions scored significantly less than cv. Imola, including all 11 accessions that supported the least *P. brassicae* sporulation. These data clearly show that quantitative resistance present in 30 diverse accessions resulted in less *P. brassicae* sporulation than in cv. Imola, which has a major QDR locus against this pathogen.

### GWA mapping of quantitative resistance against *P. brassicae*

**Fig. 2:**
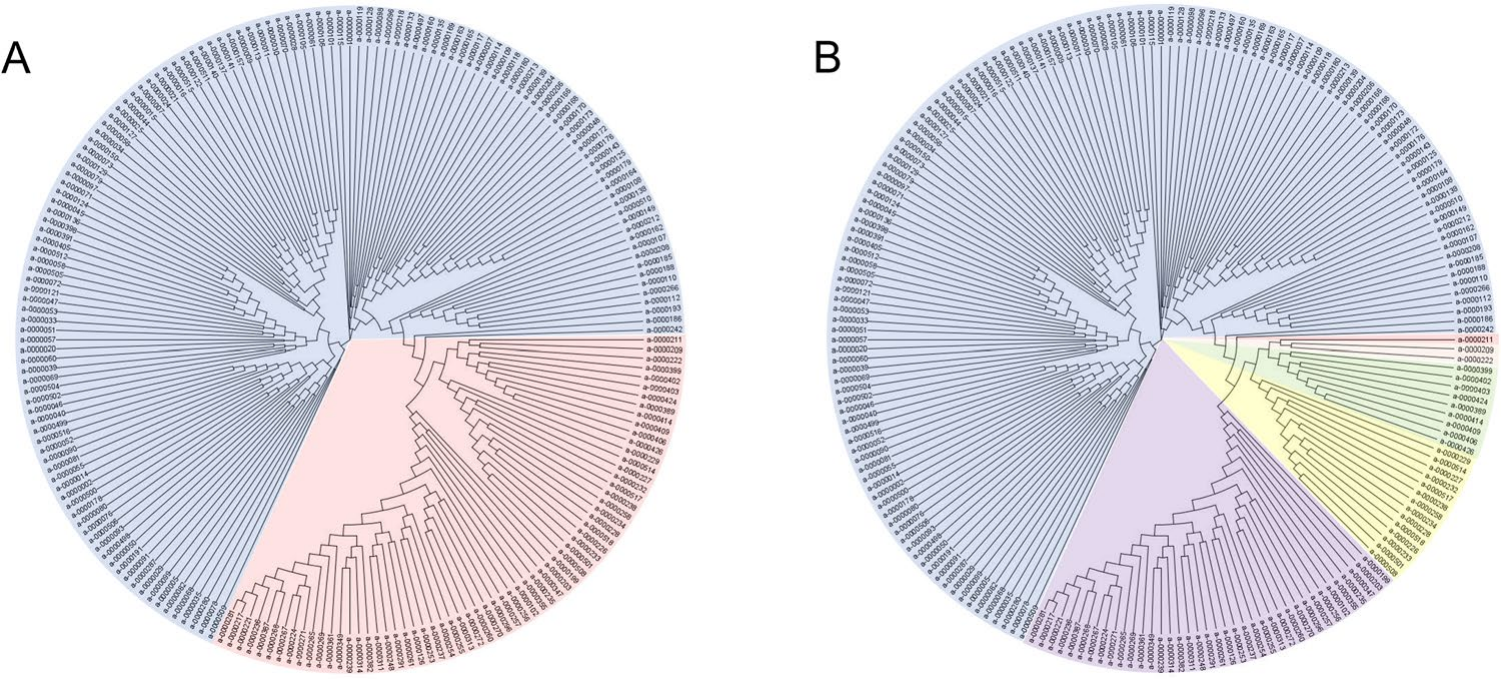
Phylogenetic tree showing the distribution of clusters from STRUCTURE analysis; **A** K = 2, divides the population into two main clusters comprising winter oilseed rape (OSR) and late winter/fodder types (blue) and other crop types (red). **B** K = 6, provides finer population structure identifying subpopulations of crop types comprising winter OSR and late winter/fodder types (blue), spring OSR (purple), semi-winter OSR (yellow), swede (green), Siberian kale group 1 (light orange), Siberian kale group 2 (red). For both A and B, some lines do not cluster phylogenetically with their crop type groups as observed in STRUCTURE due to a high degree of admixture. Detailed population structure is given in Supporting Information Figure S4 and Supporting Information Table S3

SNP data for 200 lines from the Renewable Industrial Products from Rapeseed (RIPR) genotype dataset from the resources page of York Knowledgebase (http://yorknowledgebase.info) were used for this study. Following analysis with STRUCTURE, calculation of Δ*K* divided the population into two clusters (Fig. 2); cluster one mainly comprising of winter OSR and fodder types and cluster two comprising of other crop types (Supporting Information Table S3). It was shown that *K* = 2 is a common outcome when using the Δ*K* method (Janes et al. 2017). Δ*K* frequently identifies K = 2 as the top level of hierarchical structure, and further analysis is required to determine whether more subpopulations are present. Our analysis identified a further maximum in Δ*K* at *K* = 6. This divided the population into groups comprising the different crop types; cluster one—winter and fodder; cluster two—swede; cluster three—spring OSR; clusters four and five—Siberian kale types, and six semi-winter (Chinese) OSR (Supporting Information Table S3). Subsequent phylogenetic analysis showed a delineation of these crop types (Fig. 2), with the different crop types forming clear subgroups within the tree. A number of accessions did not cluster with their given crop types. However, bar diagram outputs from STRUCTURE (Supporting Information Figure S4) showed a level of admixture within these accessions. Given the evidence for population substructure beyond *K* = 2, a structure of *K* = 6 was taken forward for use in association mapping.

Only lines with both population structure and phenotypic data were used for GWA mapping. Of the 200 lines used for generating population structure, 182 were used for GWA mapping. TASSEL identified a generalised linear modelling approach as an optimal fit for the phenotypic data (Supporting Information Figure S5). Eleven significant marker associations with LLS infection score were observed at *P* < 0.0001 (Table 1); however, none of these were significant at the FDR < 0.05. This is not unexpected as resistance against *P. brassicae* is a highly quantitative trait, with no known *R* gene loci.

**Table 1:** Genome-wide association (GWA) markers for resistance against *Pyrenopeziza brassicae* in *Brassica napus*

Locus	Brassica ID^a^	Chromosome	Marker position^b^	Reference base	Position (bp)	TAIR ID	Gene descriptor	*P* -value	−Log10P
*LLSA01*	BnaA01g25830D	A01	549	T	25,905,850	AT1G70980.1	Class II aminoacyl-tRNA and biotin synthetases superfamily protein SYNC3	1.41E−05	4.85
*LLSC01*	Bo1g123690.1	C01	471	T	37,055,696	AT3G16150.1	*N*-terminal nucleophile aminohydrolases (Ntn hydrolases) superfamily protein	1.58E−05	4.80
*LLSA09a*	Cab037694.1	A09	1371	C	175,592	AT4G00360.1	Cytochrome P450, family 86, subfamily A, polypeptide 2	1.68E−05	4.77
*LLSA02*	Cab039409.2	A02	406	C	1,016,409	AT5G06060.1	NAD(P)-binding Rossmann-fold superfamily protein	3.05E−05	4.52
*LLSC08b*	Bo8g108400.1	C08	610	G	38,642,478	AT1G12120.1	Plant protein of unknown function (DUF863)	5.24E−05	4.28
*LLSA07*	Cab020822.1	A07	852	G	27,411,592	AT1G77760.1	Nitrate reductase 1	6.29E-05	4.20
*LLSC05*	Bo5g150110.1	C05	1257	A	46,163,251	AT3G03250.1	UDP-GLUCOSE PYROPHOSPHORYLASE 1	7.16E−05	4.15
*LLSA09b*	Cab000353.1	A09	336	G	33,133,915	AT3G51370.1	Protein phosphatase 2C family protein	8.35E−05	4.08
*LLSC02*	Bo2g163990.1	C02	1694	G	51,188,221	AT5G62190.1	DEAD box RNA helicase (PRH75)	8.85E−05	4.05
*LLSC04*	Bo4g006960.1	C04	1050	A	690,146	AT3G61940.1	Cation efflux family protein (MTPA1)	9.33E−05	4.03
*LLSC08a*	Bo8g004430.1	C08	993	C	899,480	N/A	N/A	9.33E−05	4.03

^a^Markers showing association (*P* < 0.0001) with light leaf spot infection score as determined using the GLM model

^b^Position in bp given from pseudomolecule V11 (Havlickova et al. 2018)

The allelic effects of identified GWA maxima were determined (Table 2). The distribution of alleles contributing to resistance was not determined by crop type or phylogenetic relationship. Due to sequence similarities, cross alignment of transcriptome reads occurs between homeologous loci in the A and C genomes. This means that allelic calls can be the same in each genome or carry alternate alleles in the A and C genomes, referred to as a hemi-SNP, resulting in an ambiguity call during SNP calling. Three loci, *LLSC01*, *LLSA09a* and *LLSC02*, showed the strongest resistance when present as hemi-SNPs, suggesting a resistance benefit linked to carrying an alternative allele at the homeologous locus. Loci *LLSA01* and *LLSC04* showed that the majority of OSR in the panel carried the resistant allele; therefore, these may have already been selected for during breeding. Lines carrying alternate alleles at the homeologous position were also present, suggesting some breeding lines may not be optimised for these potential resistance loci. Loci *LLSA02*, *LLSA07*, *LLSA09b* and *LLSC08a* carried both A and C genome resistance alleles in a small number of lines, with most lines carrying alternate alleles at the homeologous loci. For locus *LLSC08a*, only one line carried susceptible alleles in both sub-genomes, resulting in an elevated disease score. For loci *LLSC05* and *LLSC08b*, resistant alleles at the two homeologous loci were not present within winter-OSR (WOSR) or in the case of *LLSC08b*, within the panel tested.

**Table 2:** Allelic effects of identified genome-wide association (GWA) markers for resistance against *Pyrenopeziza brassicae* in *Brassica napus*

Locus	Brassica ID	Marker position	Allele	*N*	Allelic effect	Resistant allele	Presence in WOSR^a^	Notes
*LLSA01*	BnA01g25830D	549	A/T or T/A	41	0.00	T	Common	Some accessions heterozygous, i.e. breeding potential
			TT	132	−0.62			
*LLSC01*	Bo1g123690	471	C/T or T/C	7	0.00	Mix	Rare	Unusual heterozygote advantage
			TT	66	1.31			
			CC	84	1.36			
*LLSA09a*	Cab037694	1371	G/C or C/G	12	0.00	Mix	Rare	Unusual heterozygote advantage
			CC	123	1.24			
			GG	41	1.20			
*LLSA02*	Cab039409	406	C/T or T/C	9	0.00	C	Rare	Seven WOSR accessions, but no modern ones
			TT	137	− 0.39			
			CC	32	− 1.14		Heterozygote	
*LLSC08b*	Bo8g108400	610	G/T or T/G	52	0.00	T	common	No homozygotes for T
			GG	110	0.56			
*LLSA07*	Cab020822.1	852	G/C or C/G	150	0.00	G	Rare	Nine WOSR accessions, including modern ones
			CC	12	0.67			
			GG	19	− 0.47			
*LLSC05*	Bo5g150110	1257	A/C or C/A	158	0.00	A	Rare	AA not present in WOSR
			CC	16	− 0.68			
			AA	7	− 1.44			
*LLSA09b*	Cab000353	336	G/T or T/G	133	0.00	G	Rare	Present within WOSR
			GG	48	− 0.63			
*LLSC02*	Bo2g163990	1694	AA	78	0.70	Mix	Common	Unusual heterozygote advantage
			GG	28	0.60			
			A/G or G/A	44	0.00			
*LLSC04*	Bo4g006960	1050	AA	103	−0.42	A	Common	AA common in WOSR
			GG	1	2.79			
			A/G or G/A	48	0.00			
*LLSC08a*	Bo8g004430	993	C/G or G/C	140	0.00	G	Rare	GG rare but present in two WOSR accessions
			GG	6	−0.10			
			CC	1	4.43			

^a^WOSR: Winter oilseed rape

**Fig. 3:**
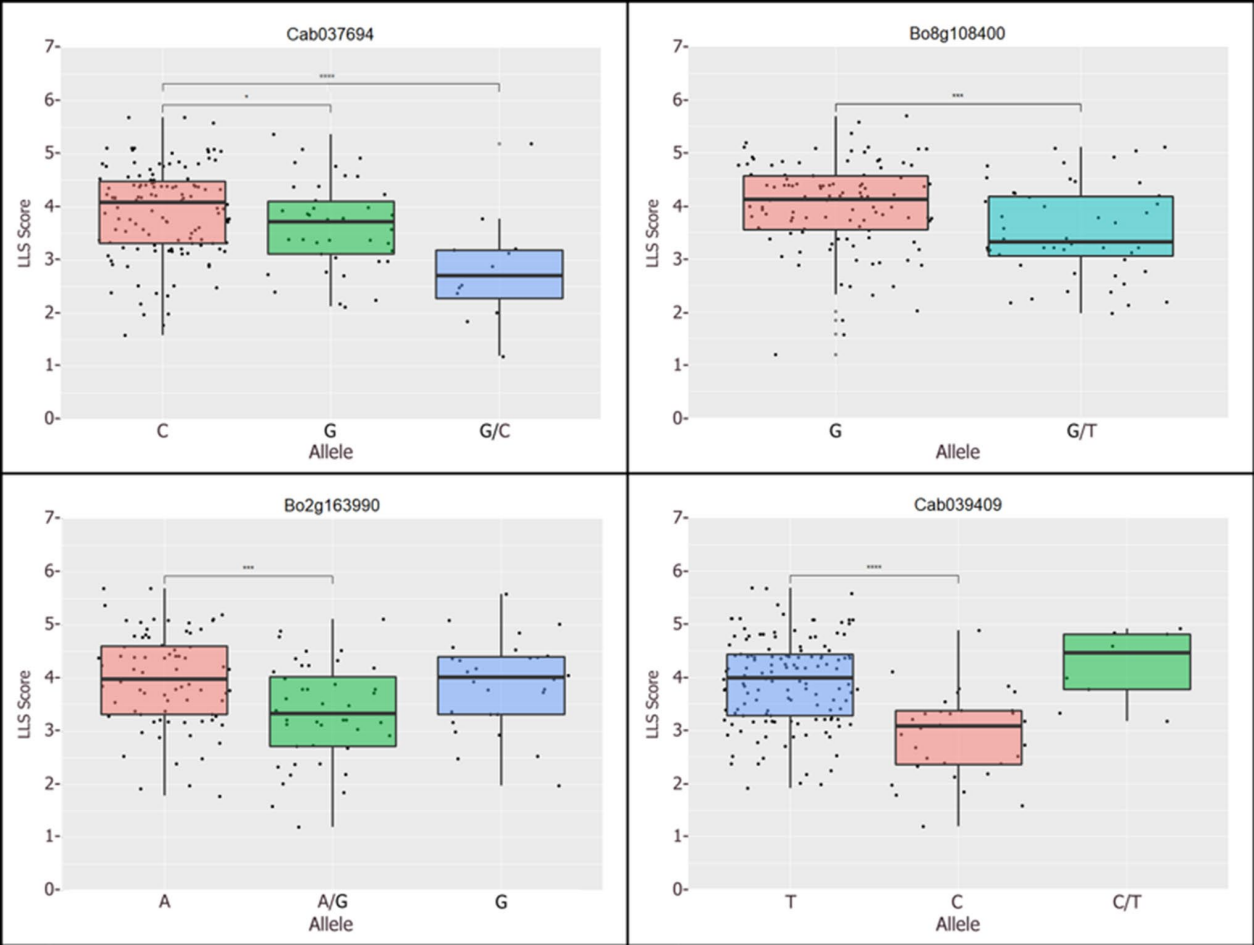
Effects of SNPs at GWA markers that were in linkage disequilibrium on QDR against *P. brassicae*. Box plots were generated in R. The box represents the lower (25%) and upper (75%) quartiles with the median shown as a bar. The whiskers extend to the most extreme data point that is no more than 1.5 times the interquartile distance from the box. Outliers may extend beyond the whiskers. Jitters illustrate individual measurements. Resistance alleles were heterozygous in three cases or homozygous in one case. Asterisks indicate significant differences at *P* < 0.05 (*), 0.001 (***) or *P* < 0.0001 (****)

Although the 11 GWA markers detail the genetic variation most significantly associated with resistance against *P. brassicae* within this analysis, transcriptome sequencing does not provide all potential genetic variants present across the panel. Genetic polymorphisms close to the causal variation will be associated on the basis of genetic linkage or LD. In the case of LD, the GWA markers may not be indicative of the causal gene. Instead, causal genes will be more or less closely linked to the GWA marker. Each GWA marker was tested for LD against all other markers on a chromosome. Four of the 11 significantly associated markers, loci *LLSA02*, *LLSA09a*, *LLSC02*, *LLSC08b*, exhibited LD with *r*^*2*^ > 0.15, thus defining the region where the causal gene is likely to be situated (Table 3, Fig. 3).

**Table 3:** Linkage disequilibrium of markers associated with resistance against *Pyrenopeziza brassicae* in *Brassica napus*

Locus	Brassica ID	Position (bp)	*P* -value	LD upper marker bound	Pseudomolecule position^a^	LD lower marker bound	Pseudomolecule position*	LD Spread (Mb)
*LLSA09*	Cab037694.1	175,592	1.68E−05	Cab037721.1	A09_000021078_000020391	Cab042612.1	A09_001395929_001397818	1.38
*LLSA02*	Cab039409.2	1,016,409	3.05E−05	Cab039419.4	A02_000954941_000946007	Cab039323.2	A02_001448258_001444400	0.49
*LLSC08*	Bo8g108400.1	38,642,478	5.24E−05	Bo8g108400.1	C08_038642478_038643814	Bo8g108420.1	C08_038666739_038667339	0.02
*LLSC02*	Bo2g163990.1	51,188,221	8.85E−05	Bo2g161630.1	C02_050925826_050930832	BnaC02g43790D	C02_052872282_052871472	1.95

^a^Position in bp given from pseudomolecule V11 (Havlickova et al. 2018)

### Eight gene expression markers are associated with resistance or susceptibility to *P. brassicae* infection

The transcriptomes of the 195 accessions were used to analyse the disease scores as a function of the expression of all gene models using a general linear model with an FDR of 0.001. A list of eight genes with *P*-values < 1.6 × 10^–7^ was selected for further analysis (Table 4). Linear regressions of the disease score versus the expression of each gene generated different slopes (Supporting Information Figure S3); the expression of seven genes was negatively correlated with the disease score. Thus, expression was greatest when the least sporulation was observed. These seven GEMs may therefore contribute to partial resistance against *P. brassicae*. Another gene, encoding an HXXXD-type acyl-transferase (Table 4), had the opposite expression pattern with an expression positively correlated with the disease score. This is therefore a candidate gene for susceptibility to *P. brassicae*.

**Table 4:** Gene expression markers (GEMs); expression of the genes listed is highly correlated with light leaf spot disease scores

Brassica ID^a^	Chromosome^b^	Log_10_P^c^	*P*-alue^d^	TAIR ID	Custom annotation^f^
Cab002134.1	A03	7.324	4.75E-08	AT4G23460.1	Adaptin β1/2-subunit of AP-1 and AP-2; sorting at endosome and TGN in conjunction with clathrin
Bo5g052100.1	C05	7.314	4.85E-08	AT2G30490.1	Cinnamate-4-hydroxylase (C4H), CYP73A5; cinnamic acid—> 4-coumaric acid; involved in lignification
Cab042707.1	A10	7.300	5.01E–08	AT5G51120.1	Cab042707 (AT5G51120): Polyadenylate-binding protein 1, controls length of poly(A) tail; localised to nuclear speckles, involved in pre-mRNA splicing
Cab000575.1	A09	6.997	1.01E–07	AT3G53990.1	Universal stress protein; localised to nucleus and cytoplasm; RNA chaperone, oxidative stress-related, antifungal activity
Bo2g028420.1	C02	6.958	1.10E−07	AT5G58700.1	Phosphatidylinositol-specific phospholipase C (PLC4); Ca2 + signalling; *in vitro* interaction with PEN3
BnaA04g20860D	A04	6.862	1.37E−07	N/A	HXXXD-type acyl-transferase
Cab046181.1	A05	6.822	1.51E−07	AT3G01090.3	SNF1 kinase homolog 10 (KIN10); stress-related energy deprivation response, autophagy activation
Bo7g093320.1	C07	6.798	1.59E−07	AT5G28060.1	40S ribosomal protein S24

^a^Pan-transcriptome gene ID, http://yorknowledgebase.info

^b^*Brassica napus* chromosome location

^c^Based on Manhattan plot analysis; false discovery rate (FDA) = 0.001

^d^Based on analysis of variance (ANOVA); disease score used as the dependent variable, gene expression (RPKM) as the independent variable

^e^The Arabidopsis Information Resource gene ID

^f^Consensus of web-based annotation and literature review

## Discussion

### QDR is the defence mechanism against *P. brassicae* in *B. napus*

Our pathosystem combined with association genetics enabled identification of candidate QDR gene loci for resistance or susceptibility to *P. brassicae*. GWA mapping clearly demonstrated the existence of multiple genes contributing to QDR, confirming that combinations of genes with relatively minor effects are the predominant mechanism of resistance against *P. brassicae*. This contrasts to resistance against *L. maculans*, which is controlled by both *R* genes (Larkan et al. 2013, 2020) and quantitative resistance loci (Huang et al. 2019). We could associate enhanced resistance with hemi-SNPs at some loci, indicating the presence of previously unknown cryptic QDR. Importantly, accessions were identified that were more resistant to *P. brassicae* than the well-characterised cv. Imola, which contains a single major QDR locus for resistance against this pathogen (Boys et al. 2012). Our investigation reveals novel QDR loci that are the primary mechanisms of resistance against *P. brassicae*. Our findings could also provide insight into mechanisms of QDR against other closely related apoplastic fungal pathosystems, including *R. commune* and *Venturia inaequalis*, for durable disease control (Stotz et al. 2014).

### Population structure analysis reflects history of OSR cultivation

The origin of cultivated OSR has been considered to be Europe (Lu et al. 2019). Spring cultivars that are commonly grown in North America and Australia were developed in the late eighteenth century, and semi-winter cultivars were introduced into China in the twentieth century. Moreover, distinct morphotypes and subspecies of swedes (*B. napus* subsp. *rapifera*) and kale (*B. napus* subsp. *pabularia*) were developed as root and leaf crops (An et al. 2019).

The population structure analysis using more than a thousand SNPs suggested genetic subdivisions into winter, spring, semi-winter and fodder OSR as well as swedes, and Siberian kale types are consistent with other published data based on *B. napus* genome sequences (An et al. 2019; Lu et al. 2019). The phylogenetic comparison reported here may be biased by the *B. napus* accessions that were used, but it suggests WOSR to be ancestral to kales, with spring OSR, swedes and semi-spring OSR being more derived (Fig. 2). The established population structure was used with associative transcriptomics to identify GWA markers and GEMs linked to quantitative resistance against *P. brassicae*.

### GWA mapping indicates four QDR loci against *P. brassicae*

GWA mapping identified four loci in LD, showing multiple markers within a region associated with the infection score, and therefore, more likely linked to QDR against *P. brassicae* than single associated SNP markers. The loci on chromosomes A02, A09, C02 and C08 were not located in regions previously identified for resistance against *P. brassicae* (Boys et al. 2012; Bradburne et al. 1999; Karandeni Dewage et al. 2022; Pilet et al. 1998). The LD observed on chromosome A09 coincides with a homeologous QTL for seed glucosinolate content (Qian et al. 2014). The spread of the LD on chromosomes A02, A09 and C02 was too large to identify candidate gene loci that might be responsible for QDR against *P. brassicae*. In contrast, the LD spread on chromosome C08 was narrow. The GWA marker Bo8g108400, corresponding to BnaC08g41550D, encodes an Cys-rich protein of unknown function. The hemi-SNP results in Gly204Cys substitution, which could be functionally significant (Perry et al. 2009). Of note, this codon is otherwise conserved in *B. oleracea*, *B. rapa* and for the homeolog *BnaA09g47370D*. Collectively, this could point to an important novel gene involved in host–pathogen interactions.

An alternative explanation could be that this gene was located right next to an ortholog of the *Arabidopsis thaliana* flavin-containing monooxygenase gene *FMO*_*GS-OX5*_ (At1g12140) that converts methylthioalkyl to methylsulfinylalkyl (MS) glucosinolates. MS glucosinolates are precursors to MS isothiocyanates that are toxic to phytopathogenic fungi (Stotz et al. 2011b). Importantly, FMO_GS-OX5_ has a preference for long-chain aliphatic glucosinolates (Li et al. 2008), which release isothiocyanates that are most toxic to phytopathogenic fungi like *S. sclerotiorum* (Stotz et al. 2011b). Notably, a mutation in the *FMO*_*GS-OX5*_ gene also altered cytokinin and jasmonate levels (Garrido et al. 2020), which is of significance considering that *P. brassicae* is a cytokinin-producing pathogen (Ashby 1997). Although the impact of jasmonates on infections by *P. brassicae* has not yet been studied, jasmonates are known to influence many pathosystems (Stotz et al. 2011a; Zheng et al. 2012). The *FMO*_*GS-OX5*_ gene is also part of an *FMO* cluster and the paralog At1g12200 that corresponds to BnaC08g41500D is induced after inoculation with *S. sclerotiorum* or *B. cinerea* (Stotz et al. 2011b). The role of this chrC08 locus in resistance against *P. brassicae* is therefore worth investigating.

The hemi-SNP for GWA marker Bo2g163990, encoding a nuclear localised DEAD box ATP-dependent RNA helicase, would result in a Gly565Asp substitution. However, in *B. rapa* and *B. napus*, a Ser and Asn are found in the same position, respectively. The amino acid substitutions would occur in the GUCT domain of these types of helicases. As these helicases are involved in development and abiotic stress responses (Liu et al. 2016; Perroud et al. 2021), it is not immediately obvious whether this helicase or another gene within LD are responsible for QDR at this locus.

The disclosed GWA markers will assist with the development of molecular markers for marker-assisted selection, plant breeding and crop improvement.

### Putative function of GEMs in QDR

The expression of eight GEMs was correlated with resistance against or susceptibility to *P. brassicae*. Amongst them were three genes that were previously reported to be involved in host pathogen interactions, including a gene encoding a cinnamate-4-hydroxylase (C4H). *C4H* genes are induced after infection of *B. napus* with *S. sclerotiorum* or *L. maculans* (Becker et al. 2017; Wu et al. 2016) and infection of the liverwort *Marchantia polymorpha* with the oomycete *Phytophthora palmivora* (Carella et al. 2019). The product 4-coumarate feeds into phenylpropanoid, flavonoid and lignin biosynthesis. Mutations in the *C4H* gene of *A. thaliana* have pleiotropic developmental defects (El Houari et al. 2021). Multiple corresponding *C4H* genes occur in *B. napus*, which may have developed specialised functions, including involvement in resistance against *P. brassicae*.

Another gene, *PLC4*, is involved in resistance against pathogens. Expression of the tomato gene *SlPLC4* is tightly regulated in response to the apoplastic fungal pathogen *Cladosporium fulvum* with a tenfold increase in expression 7 days after infection (Vossen et al. 2010). *SlPLC4* contributes to *R* gene-mediated resistance against *Cladosporium fulvum* (Vossen et al. 2010). Unlike *SlPLC6*, *SlPLC4* is not involved in resistance against *Verticillium dahliae* or *Pseudomonas syringae*. PLC4 of *A. thaliana* interacts with PEN3 (Campe et al. 2016), which is involved in resistance to penetration and export of protective metabolites against microbial invasion (Lu et al. 2015; Stein et al. 2006).

Moreover, *KIN10* recently emerged as a gene involved in resistance against the clubroot pathogen in *A. thaliana* (Chen et al. 2021). *KIN10* is a central regulator in response to energy deprivation, facilitating survival under stress conditions (Baena-Gonzalez et al. 2007) including pathogen challenge (Chen et al. 2021). These findings are consistent with the correlated of *KIN10* expression and resistance against *P. brassicae* that we observed (Supporting Information Figure S3). All of these examples suggest that the GEMs we identified are of significance and potential breeding targets. Importantly, it also implicates other, less characterised genes, in resistance against pathogens (Table 1).

## Conclusion

All resistance against *P. brassicae* found in 195 accessions of *B. napus* results from QDR with no evidence of *R* gene-mediated resistance. For genome-wide association studies, we developed an improved population structure with six phylogenetic groups based on *B. napus* crop types. We identified four gene loci significantly (*P* = 0.0001) associated with QDR and in LD. On chromosome A09, enhanced resistance was associated with a cryptic heterozygous locus for a cytochrome P450 gene co-localising with a previously described QTL for seed glucosinolate content. The expression of seven gene expression markers were positively correlated with resistance, whereas one, a HXXXD-type acyl-transferase, was negatively correlated and so is a potential susceptibility gene. The results provide new insight into QDR against *P. brassicae* in *B. napus* and can be used for marker-assisted breeding in crop improvement.

## Supplementary Information

Below is the link to the electronic supplementary material.

Supplementary file1 (JPG 2508 KB)

Supplementary file2 (JPG 436 KB)

Supplementary file3 (JPG 1057 KB)

Supplementary file4 (JPG 570 KB)

Supplementary file5 (PDF 89 KB)

Supplementary file6 (PDF 74 KB)

## Data Availability

The datasets generated during and/or analysed during the current study are available from the corresponding author on reasonable request.
