# Supplementary figures and images for: Novel gene loci associated with susceptibility or cryptic quantitative resistance to Pyrenopeziza brassicae in Brassica napus
Source: Theor Appl Genet. 2023 Mar 23;136(4):71. doi: 10.1007/s00122-023-04243-y (PMC10036280; doi:10.1007/s00122-023-04243-y)

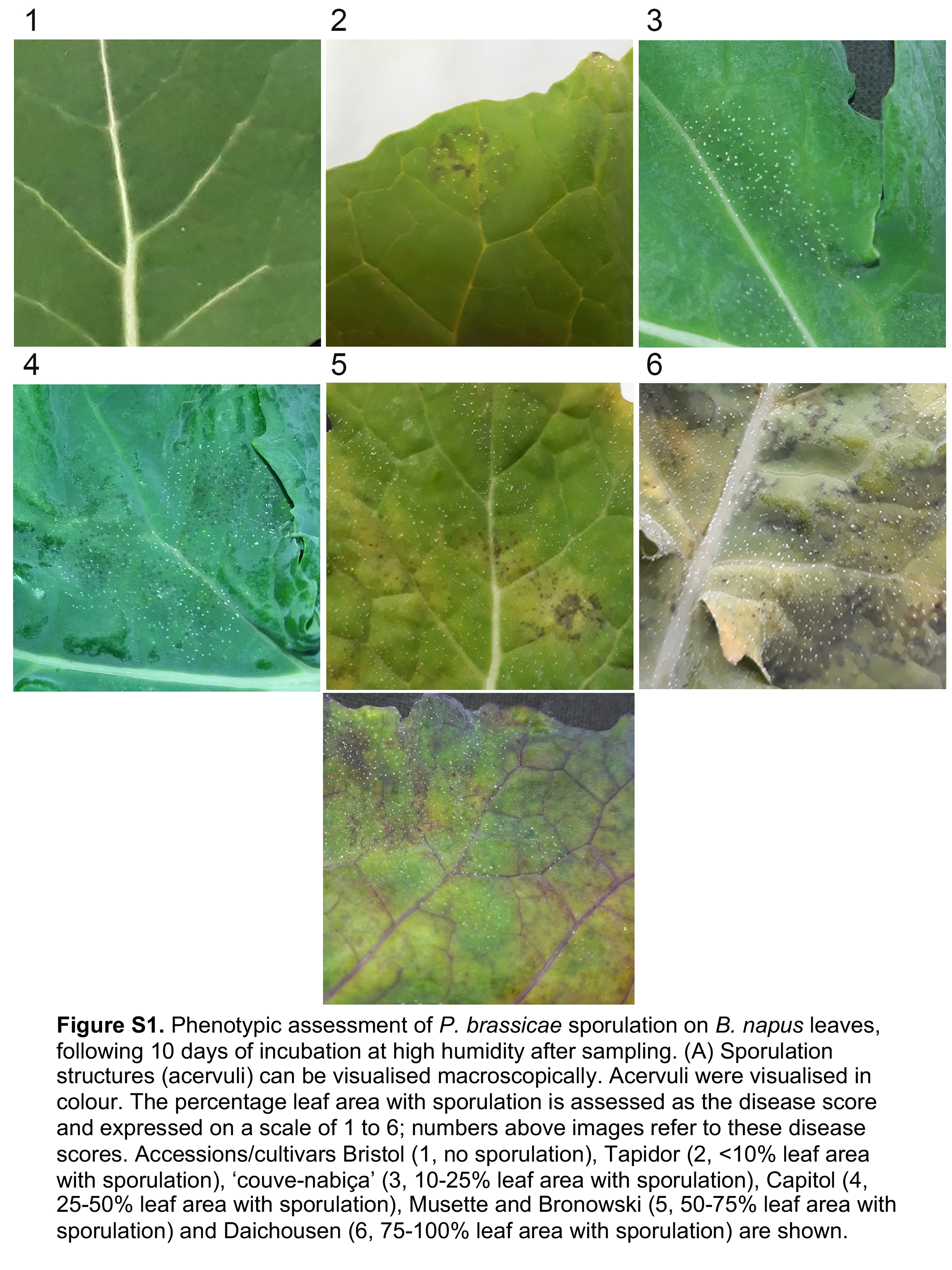

Supplement: Supplementary file 1 — Supplementary file1 (JPG 2508 KB) [file 122_2023_4243_MOESM1_ESM.jpg]

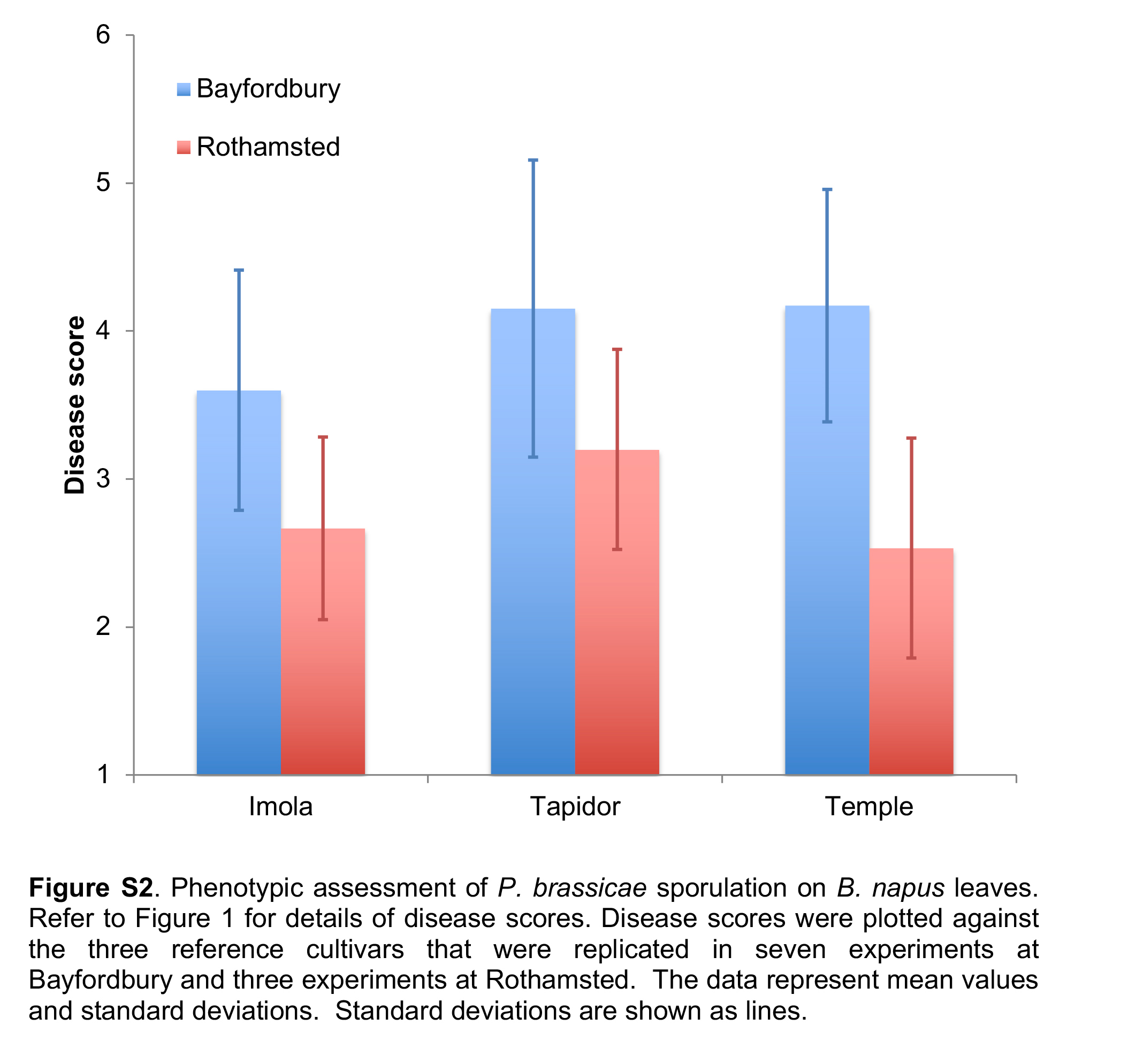

Supplement: Supplementary file 2 — Supplementary file2 (JPG 436 KB) [file 122_2023_4243_MOESM2_ESM.jpg]

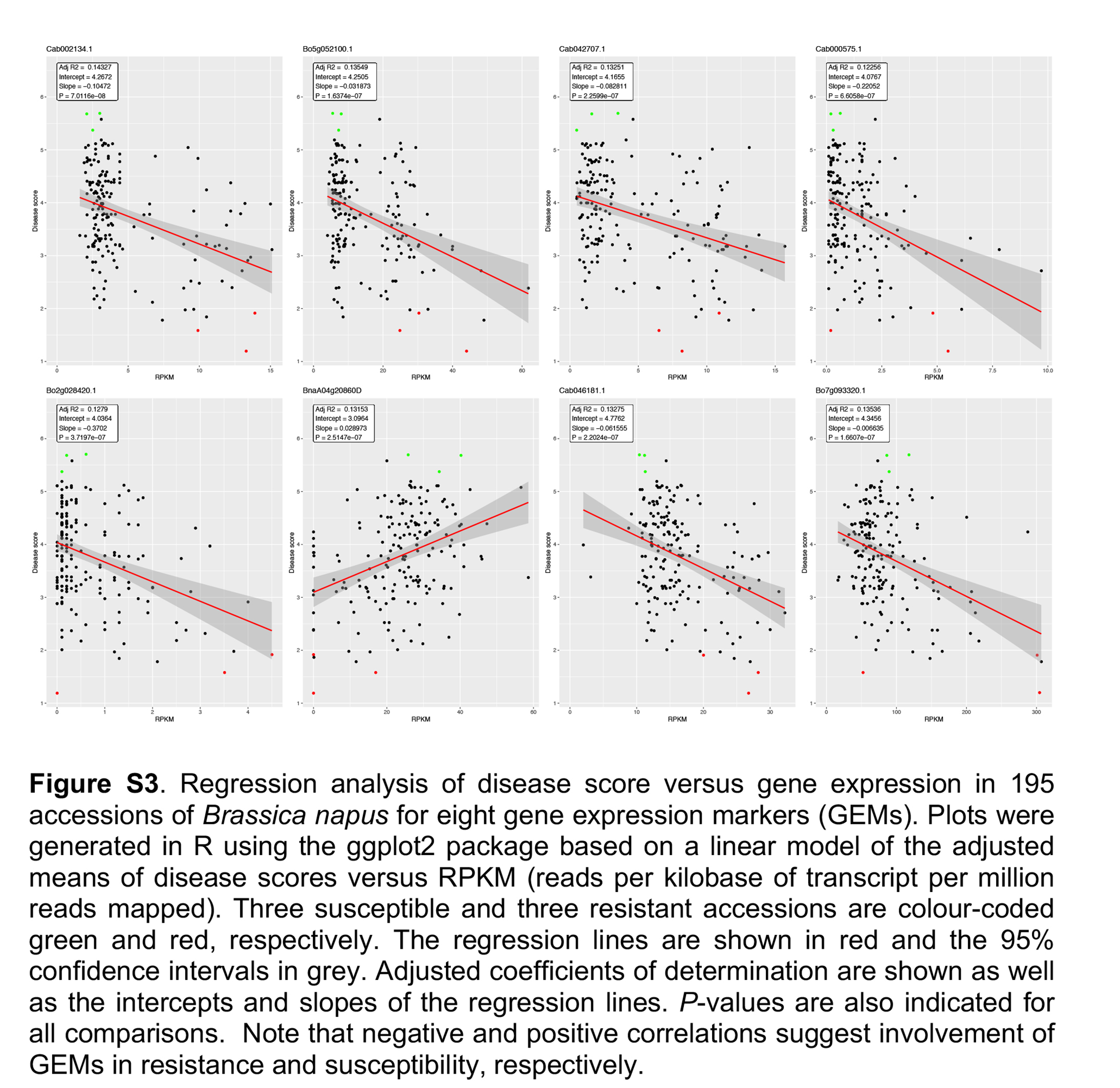

Supplement: Supplementary file 3 — Supplementary file3 (JPG 1057 KB) [file 122_2023_4243_MOESM3_ESM.jpg]

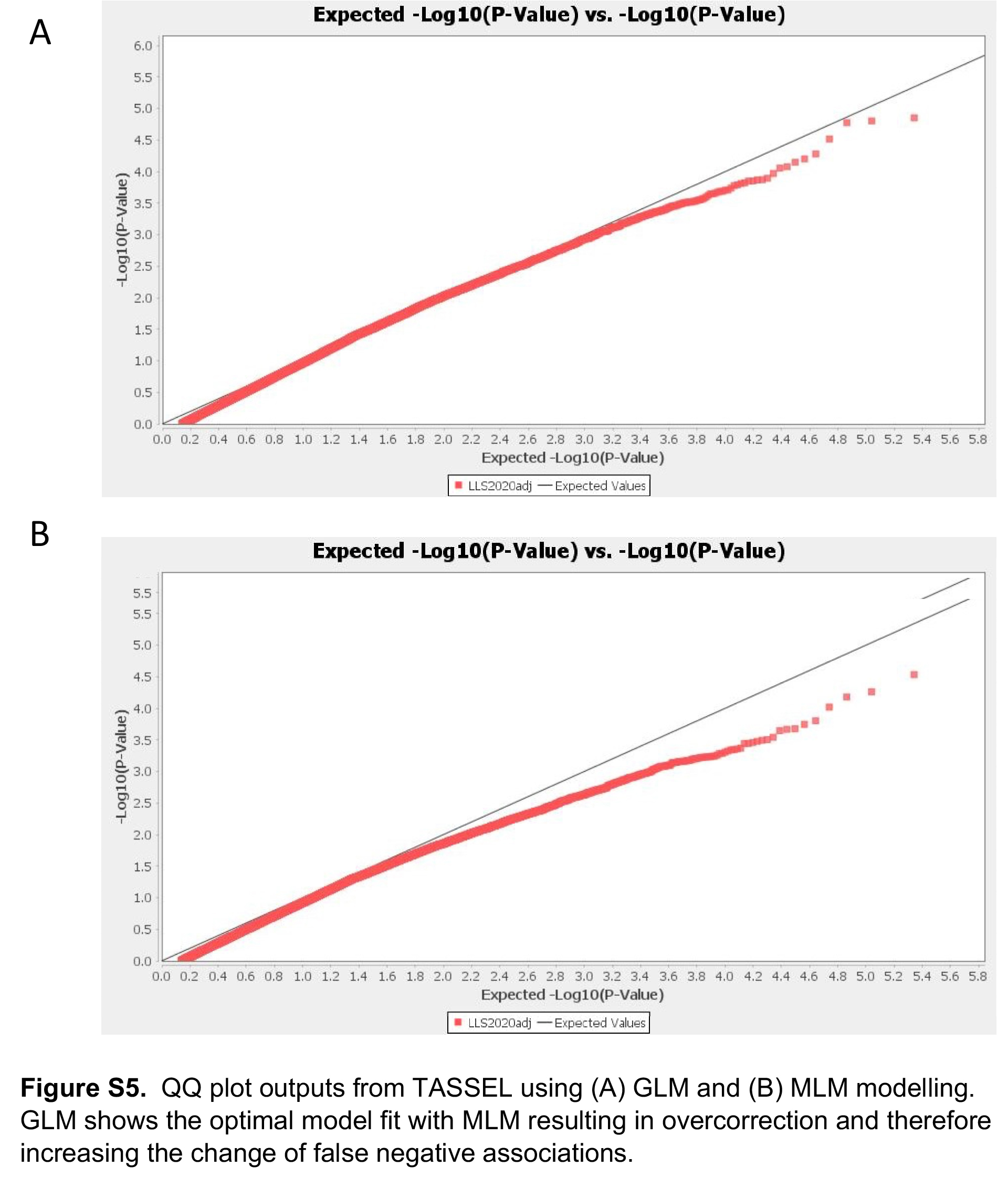

Supplement: Supplementary file 4 — Supplementary file4 (JPG 570 KB) [file 122_2023_4243_MOESM4_ESM.jpg]
