# Supplementary material for: Novel gene loci associated with susceptibility or cryptic quantitative resistance to Pyrenopeziza brassicae in Brassica napus
Source: Theor Appl Genet. 2023 Mar 23;136(4):71. doi: 10.1007/s00122-023-04243-y (PMC10036280; doi:10.1007/s00122-023-04243-y)
Supplement: Supplementary file 5 — Supplementary file5 (PDF 89 KB) [file 122_2023_4243_MOESM5_ESM.pdf]

Table S2. Accessions ranked for disease score, i.e. *Pyrenopeziza brassicae* sporulation, on a scale of 1 to 6 observed under glasshouse conditions.

Accession	Crop type	Experiment[1]	Score[2]
Cubs Root	Spring OSR	5	1.19
Posh	Modern Winter OSR	2	1.53
Dwarf Essex	Forage Rape	6	1.58
Jaune a Collet Vert	Swede	6	1.78
SWU Chinese 1	Semiwinter OSR	10	1.84
Altasweet	Swede	3	1.91
Liho	Spring Fodder	7	1.97
Drummonds Purple Top	Swede	6	1.98
Akela	Winter OSR	4	2.01
Tribune	Spring OSR	8	2.12
Q100	Synthetic	2	2.17
Furax	Spring OSR	6	2.18
Zenith	Winter OSR	10	2.24
Zairai Chousenshu	Spring OSR	8	2.32
Kromerska	Modern Winter OSR	7	2.37
Janetzkis Schlesischer	Winter OSR	6	2.38
Vige DH	Swede	8, 10	2.38
Chuanyou 2	Semiwinter OSR	5	2.39
Pacific	Modern Winter OSR	3	2.48
Resyn-H048	Winter OSR	9	2.48
Odin	Spring OSR	1	2.52
Omega	Spring OSR	2	2.52
Weihenstephaner	Spring OSR	8	2.52
Slapska, Slapy	Winter OSR	9	2.68
Yudal	Spring OSR	3	2.71
Willi	Spring OSR	8	2.72
Lipid	Modern Winter OSR	7	2.77
Prince	Modern Winter OSR	3	2.88
Rodeo	Modern Winter OSR	9	2.88
SLM 0413	Modern Winter OSR	9	2.88
Matador	Winter OSR	3	2.91
Tina	Swede	3	2.91
Tantal	Spring OSR	8	2.92
TN172DH	Semiwinter x Winter OSR	1	2.93
N01D-1330	Spring OSR	7	2.97
Siberische Boerenkool	Siberian Kale	2	2.97
Binera	Winter Fodder	5	2.99
SWU Chinese 5	Semiwinter OSR	10	3.04
SLM 0512	Modern Winter OSR	9	3.08
Karoon-057DH	Spring OSR	3	3.11
Sensation NZ	Swede	3	3.11
Rucabo	Spring OSR	8	3.12
Ningyou7	Semiwinter OSR	2	3.13

Pollen	Winter OSR	9, 10	3.16
Ceska Krajova	Spring OSR	2	3.17
Magma	Spring OSR	7	3.17
Mansholt	Winter OSR	7	3.17
Milena	Modern Winter OSR	7	3.17
Stellar DH	Spring OSR	2	3.17
Evvin	Winter OSR	6	3.18
Bangholm PT	Swede	5	3.19
Bienvenu DH4	Winter OSR	4	3.21
Lembkes Malchower (Lenora)	Winter OSR	4	3.21
Norin	Winter OSR	4	3.21
Palmedor	Winter OSR	1	3.21
Vision	Winter OSR	4	3.21
Zephir	Modern Winter OSR	10	3.24
Orlando	Modern Winter OSR	1	3.28
Roxet	Modern Winter OSR	9	3.28
Slovenska Krajova	Winter OSR	3	3.31
Imola[3]	Winter OSR	1 - 10	3.32
Mohican	Winter OSR	7	3.32
Mytnickij	Winter OSR	7	3.32
Pobeda	Modern Winter OSR	3	3.32
Westar DH	Spring OSR	1, 3	3.32
Taisetsu	Winter Vegetable	1	3.33
TN145	Semiwinter x Winter OSR	1	3.33
Bronowski	Spring OSR	2	3.37
Drakkar	Spring OSR	2	3.37
Krapphauser	Winter OSR	7	3.37
Gross-Luesewitzer	Winter OSR	6	3.38
Helios	Spring OSR	6	3.38
Amor	Modern Winter OSR	5	3.39
Brutor	Spring OSR	5	3.39
York	Swede	4	3.41
Panter	Winter OSR	2	3.48
Baltia	Winter OSR	3	3.51
Caramba	Modern Winter OSR	5	3.54
Erglu	Spring OSR	2	3.57
Verona	Winter OSR	2	3.57
Zhongshuang II	Semiwinter OSR	2	3.57
Karat	Spring OSR	6	3.58
Coriander	Winter OSR	4	3.61
Temple[3]	Winter OSR	1 - 10	3.68
Hansen x Gaspard DH line	Winter OSR	3	3.71
Nemertschanskij 1	Winter OSR	2	3.72
Surpass 400-024DH	Spring OSR	8	3.72
Canard	Winter Forage Rape	1	3.73
Ladoga	Modern Winter OSR	7	3.77

LSF 0519	Modern Winter OSR	7	3.77
Mazowiecki	Spring OSR	7	3.77
N02D-1952	Spring OSR	1	3.77
Emerald	Winter OSR	6	3.78
Falcon	Modern Winter OSR	6	3.78
Jessica	Modern Winter OSR	6	3.78
Lesira	Winter OSR	1	3.78
Cresor	Spring OSR	5	3.79
Diamant	Winter OSR	5	3.79
SWU Chinese 2	Semiwinter OSR	10	3.84
SWU Chinese 8	Semiwinter OSR	10	3.84
Tapidor DH[3]	Winter OSR	1 - 10	3.87
Norde	Winter OSR	4	3.88
Phil	Winter OSR	4	3.88
Hearty	Modern Winter OSR	1	3.93
Monty-028DH	Spring OSR	7	3.93
Quinta	Winter OSR	1	3.93
Couve-Nabiça	Leafy Vegetable	2	3.97
Lisabeth	Modern Winter OSR	7	3.97
Shengliyoucai	Chinese	2	3.97
Amber x Commanche	Winter OSR	5	3.99
Askari	Winter OSR	5	3.99
Bristol[3]	Modern Winter OSR	1 - 7	3.99
Canberra x Courage DH line	Winter OSR	5	3.99
Sobotkowski	Winter OSR	10	4.04
Remy	Modern Winter OSR	9	4.08
Apex-93_5 x Ginyou_3 DH line	Winter OSR	3	4.11
Topas	Spring OSR	3	4.11
NK Bravour	Modern Winter OSR	1	4.12
Licrown x Express DH line	Winter OSR	7	4.17
Lipton	Modern Winter OSR	7	4.17
Lirabon	Winter OSR	7	4.17
Loras	Spring OSR	7	4.17
Express 617	Modern Winter OSR	6	4.18
Flip	Winter OSR	6	4.18
Fortis	Modern Winter OSR	6	4.18
Cobra	Winter OSR	5	4.19
Rocket	Winter OSR	4	4.21
Smart	Modern Winter OSR	10	4.24
Sollux	Winter OSR	10	4.24
SWU Chinese 9	Semiwinter OSR	10	4.24
Viking	Modern Winter OSR	10	4.24
Wilhelmsburger DH	Swede	3	4.31
Excel	Modern Winter OSR	1	4.33
JetNeuf	Winter OSR	1	4.33
Ramses	Winter OSR	1	4.33

Agalon	Modern Winter OSR	4	4.35
Groene Groninger Snijmoes	Siberian Kale	2	4.37
Kvintett	Winter OSR	7	4.37
Madora	Winter OSR	7	4.37
Maplus	Winter OSR	7	4.37
Xiangyou 15	Semiwinter OSR	2	4.37
Doral	Winter OSR	6	4.38
Erake	Spring OSR	6	4.38
Beluga	Modern Winter OSR	5	4.39
Boston	Modern Winter OSR	5	4.39
Catana	Winter OSR	5	4.39
Contact	Modern Winter OSR	5	4.39
Alesi	Modern Winter OSR	4	4.41
Dippes	Winter OSR	4	4.41
Huron x Navajo	Winter OSR	4	4.41
Inca x Contact	Winter OSR	4	4.41
SWGospel	Modern Winter OSR	10	4.44
Aragon	Modern Winter OSR	5	4.46
Brauner Schnittkohl	Siberian Kale	3	4.51
POH 285, Bolko	Winter OSR	4	4.51
Eurol	Winter OSR	1	4.53
Madrigal x Recital DH line	Winter OSR	1	4.53
Rapid Cycling Rape (CrGC5)	Spring OSR	2	4.57
Expert	Winter OSR	6	4.58
Idol	Modern Winter OSR	6	4.58
Daichousen (mizuyasu)	Spring OSR	5	4.59
Castille	Winter OSR	4	4.61
Aberdeenshire Prize	Swede	3	4.71
Apex	Winter OSR	1, 2	4.75
Abukuma Natane	Winter OSR	2	4.77
Duplo	Spring OSR	2	4.77
Leopard	Winter OSR	7	4.77
Falstaff	Winter OSR	6	4.78
Ability	Spring OSR	4	4.81
Cabernet	Winter OSR	4	4.81
Samourai	Winter OSR	4	4.81
Shannon x Winner DH line	Winter OSR	4	4.81
V8	Winter OSR	10	4.84
Wild Accession	Wild Accession	10	4.84
Capitol	Winter OSR	1, 2	4.85
Oase	Modern Winter OSR	3	4.88
Ragged Jack	Siberian Kale	8, 10	4.88
Musette	Modern Winter OSR	3	4.92
Tribute	Spring OSR	8	4.92
Alku	Spring OSR	4	5.01
SWU Chinese 3	Semiwinter OSR	10	5.04

Vinnickij 15/59	Winter OSR	10	5.04
Pirola	Modern Winter OSR	4	5.08
Rapid	Modern Winter OSR	9	5.08
Sarepta	Winter OSR	9	5.08
Savannah	Modern Winter OSR	9	5.08
Fortin Family	Swede	3	5.11
Huguenot	Swede	3	5.11
Rafal DH line	Winter OSR	1	5.11
Svalöf's Gulle	Spring OSR	8	5.12
Dimension	Winter OSR	5	5.19
Laser	Modern Winter OSR	7	5.37
Moana, Moana Rape	Fodder Rape	8, 10	5.58
Sansibar	Modern Winter OSR	9	5.68
Cabriolet[3]	Winter OSR	1, 8 - 10	5.69

[1] Experiments 1 - 7 were done at a Bayfordbury glasshouse of the University of Hertfordshire. Experiments 8 - 10 were done at a Rothamsted Research glasshouse. *P. brassicae* populations were obtained from field sites on the island of Fehmarn in Germany and at Rothamsted Research in 2016 and 2019 for the Bayfordbury and Rothamsted Research experiments, respectively. For details of temperatures during experiments, see Table S1.

[2] Light leaf spot disease was scored on a scale of 1 to 6, with scores of 1 and 6 supporting least and most fungal sporulation (Fig. 1). The data represent a total of 1190 assessments that were collected in 10 experiments. Data points are adjusted means of ≥ 5 replicates per accession after non-linear mixed model analysis.

[3] Imola (resistant), Tapidor and Temple were used as reference cultivars in all 10 experiments. The susceptible cultivars were Bristol in experiments 1-7 and Cabriolet in experiments 8-10.
