# Supplementary material for: Novel gene loci associated with susceptibility or cryptic quantitative resistance to Pyrenopeziza brassicae in Brassica napus
Source: Theor Appl Genet. 2023 Mar 23;136(4):71. doi: 10.1007/s00122-023-04243-y (PMC10036280; doi:10.1007/s00122-023-04243-y)
Supplement: Supplementary file 6 — Supplementary file6 (PDF 74 KB) [file 122_2023_4243_MOESM6_ESM.pdf]

Table S3. Detailes of Brassica napus accessions used for STRUCTURE analysis and their cluster membership at K = 2 and K = 6

STRUCTURE accession number	Transcriptome source code	Accession	Crop type	Cluster K=2	Cluster K=6
1	a-0000001	Alesi	Modern Winter OSR	1	1
2	a-0000002	Remy	Modern Winter OSR	1	1
3	a-0000005	Pirola	Modern Winter OSR	1	1
4	a-0000007	Milena	Modern Winter OSR	1	1
5	a-0000009	Agalon	Modern Winter OSR	1	1
6	a-0000011	Picasso	Modern Winter OSR	1	1
7	a-0000014	Rodeo	Modern Winter OSR	1	1
8	a-0000015	Rapid	Modern Winter OSR	1	1
9	a-0000016	Boston	Modern Winter OSR	1	1
10	a-0000020	Pacific	Modern Winter OSR	1	1
11	a-0000021	Savannah	Modern Winter OSR	1	1
12	a-0000024	Ladoga	Modern Winter OSR	1	1
13	a-0000025	Atlantic	Modern Winter OSR	1	1
15	a-0000029	Idol	Modern Winter OSR	1	1
16	a-0000030	Vivol	Modern Winter OSR	1	1
17	a-0000033	Lisabeth	Modern Winter OSR	1	1
18	a-0000034	Lipid	Modern Winter OSR	1	1
19	a-0000035	Lipton	Modern Winter OSR	1	1
20	a-0000037	Contact	Modern Winter OSR	1	1
21	a-0000039	Oase	Modern Winter OSR	1	1
23	a-0000044	Laser	Modern Winter OSR	1	1
24	a-0000045	Fortis	Modern Winter OSR	1	1
25	a-0000046	Smart	Modern Winter OSR	1	1
26	a-0000047	Roxet	Modern Winter OSR	1	1
27	a-0000048	NK Bravour	Modern Winter OSR	1	1
28	a-0000050	Aviso	Modern Winter OSR	1	1
29	a-0000051	Sansibar	Modern Winter OSR	1	1
30	a-0000052	SWGospel	Modern Winter OSR	1	1
33	a-0000056	Musette	Modern Winter OSR	1	1
36	a-0000060	Viking	Modern Winter OSR	1	1
37	a-0000061	Aragon	Modern Winter OSR	1	1
38	a-0000068	Zephir	Modern Winter OSR	1	1
39	a-0000069	SLM 0413	Modern Winter OSR	1	1
40	a-0000070	SLM 0512	Modern Winter OSR	1	1
41	a-0000071	LSF 0519	Modern Winter OSR	1	1
42	a-0000072	Beluga	Modern Winter OSR	1	1
43	a-0000073	Amor	Modern Winter OSR	1	1
44	a-0000076	Caramba	Modern Winter OSR	1	1
45	a-0000078	Express 617	Modern Winter OSR	1	1
46	a-0000079	Jessica	Modern Winter OSR	1	1
47	a-0000080	Orlando	Modern Winter OSR	1	1
49	a-0000082	Prince	Modern Winter OSR	1	1
90	a-0000150	Kromerska	Modern Winter OSR	1	1
166	a-0000355	Pobeda	Modern Winter OSR	1	3
14	a-0000028	Capitol	Winter OSR	1	1
22	a-0000040	Apex	Winter OSR	1	1
31	a-0000053	Verona	Winter OSR	1	1
32	a-0000055	Expert	Winter OSR	1	1
34	a-0000057	Kvintett	Winter OSR	1	1
35	a-0000058	Falstaff	Winter OSR	1	1
48	a-0000081	Pollen	Winter OSR	1	1
50	a-0000090	Apex-93_5 X Ginyou_3 DH line	Winter OSR	1	1
51	a-0000091	Bienvenu DH4	Winter OSR	1	1
52	a-0000093	Canberra x Courage DH line	Winter OSR	1	1
53	a-0000096	Hansen X Gaspard DH line	Winter OSR	1	1

54	a-0000097	Madrigal x Recital DH line	Winter OSR	1	1
55	a-0000098	Rafal DH line	Winter OSR	1	1
56	a-0000099	Tapidor DH	Winter OSR	1	1
57	a-0000101	Eurol	Winter OSR	1	1
59	a-0000105	Licrown X Express DH line	Winter OSR	1	1
60	a-0000106	Shannon x Winner DH line	Winter OSR	1	1
61	a-0000107	Janetzkis Schlesischer	Winter OSR	1	1
62	a-0000108	Lembkes Malchower (Lenora)	Winter OSR	1	1
63	a-0000109	Norin	Winter OSR	1	1
64	a-0000110	Olimpiade	Winter OSR	1	1
65	a-0000112	Mansholt	Winter OSR	1	1
66	a-0000113	Samourai	Winter OSR	1	1
67	a-0000114	Sollux	Winter OSR	1	1
68	a-0000115	Akela	Winter OSR	1	1
69	a-0000117	Maplus	Winter OSR	1	1
70	a-0000118	Askari	Winter OSR	1	1
71	a-0000119	Lirabon	Winter OSR	1	1
72	a-0000121	JetNeuf	Winter OSR	1	1
73	a-0000122	Cobra	Winter OSR	1	1
74	a-0000124	Mohican	Winter OSR	1	1
75	a-0000125	Flip	Winter OSR	1	1
77	a-0000127	Phil	Winter OSR	1	1
78	a-0000128	Leopard	Winter OSR	1	1
80	a-0000133	Baltia	Winter OSR	1	1
81	a-0000135	Brink	Winter OSR	1	1
82	a-0000136	Ceres	Winter OSR	1	1
83	a-0000137	Coriander	Winter OSR	1	1
84	a-0000138	Diamant	Winter OSR	1	1
85	a-0000139	Dippes	Winter OSR	1	1
86	a-0000140	Doral	Winter OSR	1	1
87	a-0000141	Edita	Winter OSR	1	1
88	a-0000143	Gross-Luesewitzer	Winter OSR	1	1
89	a-0000149	Krapphauser	Winter OSR	1	1
91	a-0000157	Madora	Winter OSR	1	1
92	a-0000160	Matador	Winter OSR	1	1
93	a-0000162	Moldavia	Winter OSR	1	1
94	a-0000163	Mytnickij	Winter OSR	1	1
95	a-0000164	Nemertschanskij 1	Winter OSR	1	1
96	a-0000165	Norde	Winter OSR	1	1
97	a-0000166	Panter	Winter OSR	1	1
98	a-0000168	Ramses	Winter OSR	1	1
99	a-0000169	Sarepta	Winter OSR	1	1
100	a-0000170	Skrzeszowicki	Winter OSR	1	1
101	a-0000172	Slovenska Krajova	Winter OSR	1	1
102	a-0000173	Sobotkowski	Winter OSR	1	1
103	a-0000176	Trebicska	Winter OSR	1	1
104	a-0000178	Vinnickij 15/59	Winter OSR	1	1
105	a-0000179	Wolynski	Winter OSR	1	1
106	a-0000180	V8	Winter OSR	1	1
109	a-0000188	Emerald	Winter OSR	1	1
119	a-0000212	Slapska, Slapy	Winter OSR	1	1
120	a-0000213	Abukuma Natane	Winter OSR	1	1
182	a-0000497	Cabernet	Winter OSR	1	1
183	a-0000498	Cabriolet	Winter OSR	1	1
184	a-0000499	Castille	Winter OSR	1	1
185	a-0000500	Catana	Winter OSR	1	1
187	a-0000502	Dimension	Winter OSR	1	1
188	a-0000504	Flash	Winter OSR	1	1

189	a-0000505	Huron x Navajo	Winter OSR	1	1
190	a-0000506	Inca x Contact	Winter OSR	1	1
192	a-0000509	Palmedor	Winter OSR	1	1
193	a-0000510	POH 285, Bolko	Winter OSR	1	1
194	a-0000511	Quinta	Winter OSR	1	1
195	a-0000512	Rocket	Winter OSR	1	1
197	a-0000515	Temple	Winter OSR	1	1
198	a-0000516	Vision	Winter OSR	1	1
114	a-0000204	Q100	Synthetic	1	1
108	a-0000186	Moana, Moana Rape	Fodder Rape	1	1
111	a-0000193	Dwarf Essex	Forage Rape	1	1
110	a-0000191	Binera	Winter Fodder	1	1
112	a-0000199	Parapluie	Winter Fodder	1	1
107	a-0000185	Canard	Winter Forage Rape	1	1
115	a-0000206	Brauner Schnittkohl	Siberian Kale	1	1
122	a-0000218	Groene Groninger Snijmoes	Siberian Kale	1	1
171	a-0000391	Fortin Family	Swede	1	1
172	a-0000398	Huguenot	Swede	1	1
176	a-0000405	Aberdeenshire Prize	Swede	1	1
138	a-0000242	Cresor	Spring OSR	1	1
149	a-0000266	Tantal	Spring OSR	1	1
156	a-0000280	Topas	Spring OSR	1	1
158	a-0000287	Ability	Spring OSR	1	1
133	a-0000235	Drakkar	Spring OSR	1	3
129	a-0000229	SWU Chinese 5	Semiwinter OSR	2	1
116	a-0000208	couve-nabiça	Leafy Vegetable	2	1
113	a-0000203	Taisetsu	Winter vegetable	2	1
170	a-0000389	Vige DH	Swede	2	2
173	a-0000399	Jaune a Collet Vert	Swede	2	2
174	a-0000402	Sensation NZ	Swede	2	2
175	a-0000403	Wilhelmsburger DH	Swede	2	2
177	a-0000406	Altasweet	Swede	2	2
178	a-0000409	Bangholm PT	Swede	2	2
179	a-0000414	Drummonds Purple Top	Swede	2	2
180	a-0000424	Tina	Swede	2	2
181	a-0000426	York	Swede	2	2
58	a-0000102	Lesira	Winter OSR	2	3
76	a-0000126	Zenith	Winter OSR	2	3
121	a-0000217	Evvin	Winter OSR	2	3
123	a-0000221	Rapid Cycling Rape (CrGC5)	Spring OSR	2	3
125	a-0000224	Wild Accession	Wild accession	2	3
134	a-0000236	Stellar DH	Spring OSR	2	3
135	a-0000237	Westar DH	Spring OSR	2	3
137	a-0000239	Brutor	Spring OSR	2	3
139	a-0000248	Karat	Spring OSR	2	3
140	a-0000253	Karoo-057DH	Spring OSR	2	3
141	a-0000254	Monty-028DH	Spring OSR	2	3
142	a-0000255	N01D-1330	Spring OSR	2	3
143	a-0000256	N02D-1952	Spring OSR	2	3
144	a-0000257	Surpass 400-024DH	Spring OSR	2	3
146	a-0000260	Erglu	Spring OSR	2	3
147	a-0000261	Helios	Spring OSR	2	3
148	a-0000265	Mazoweicki	Spring OSR	2	3
150	a-0000267	Weihenstephaner	Spring OSR	2	3
151	a-0000268	Liho	Spring Fodder	2	3
152	a-0000269	Alku	Spring OSR	2	3
153	a-0000270	Bronowski	Spring OSR	2	3
154	a-0000271	Ceska Krajova	Spring OSR	2	3

155	a-0000272	Duplo	Spring OSR	2	3
157	a-0000281	Tribute	Spring OSR	2	3
159	a-0000291	Magma	Spring OSR	2	3
160	a-0000296	Tribune	Spring OSR	2	3
161	a-0000311	Daichousen (mizuyasu)	Spring OSR	2	3
162	a-0000313	Erake	Spring OSR	2	3
163	a-0000314	Furax	Spring OSR	2	3
164	a-0000347	Odin	Spring OSR	2	3
165	a-0000349	Omega	Spring OSR	2	3
167	a-0000361	Rucabo	Spring OSR	2	3
168	a-0000382	Willi	Spring OSR	2	3
169	a-0000387	Zairai Chousenshu	Spring OSR	2	3
124	a-0000222	Russain Kale	Siberian Kale	2	4
117	a-0000209	Ragged Jack	Siberian Kale	2	4
118	a-0000211	Siberische Boerenkool	Siberian Kale	2	5
79	a-0000129	RESYN-H048	Winter OSR	2	5
126	a-0000226	SWU Chinese 1	Semiwinter OSR	2	6
127	a-0000227	SWU Chinese 2	Semiwinter OSR	2	6
128	a-0000228	SWU Chinese 3	Semiwinter OSR	2	6
130	a-0000232	SWU Chinese 8	Semiwinter OSR	2	6
131	a-0000233	SWU Chinese 9	Semiwinter OSR	2	6
132	a-0000234	Zhouyou	Semiwinter OSR	2	6
186	a-0000501	Chuanyou 2	Semiwinter OSR	2	6
191	a-0000508	Ningyou7	Semiwinter OSR	2	6
196	a-0000514	Shengliyoucai	Semiwinter OSR	2	6
199	a-0000517	Xiangyou 15	Semiwinter OSR	2	6
200	a-0000518	Zhongshuang II	Semiwinter OSR	2	6
136	a-0000238	Yudal	Spring OSR	2	6
145	a-0000258	Cubs Root	Spring OSR	2	6
